# 30-day outcomes of robotic versus laparoscopic Heller myotomy

**DOI:** 10.1007/s00464-025-12085-6

**Published:** 2025-08-29

**Authors:** Paul J. Brosnihan, Ashkan Moazzez, Junko J. Ozao-Choy, Christian Perez, Amy K. Yetasook

**Affiliations:** 1 Harbor UCLA Medical Center, 1000 W Carson Street, Box 461, Torrance, CA 90509 USA; 2 Department of Surgery, Harbor UCLA Medical Center, 1000 W. Carson Street, Torrance, CA 90502 USA

**Keywords:** Achalasia, Heller myotomy, MIS, Robotic surgery

## Abstract

**Introduction:**

Minimally invasive Heller myotomy has become the standard of care to treat patients with esophageal achalasia with improved morbidity and mortality compared to its open counterpart; however few studies have prospectively compared Robotic Heller myotomy (RAHM) to laparoscopic Heller myotomy (LHM).

**Methods:**

The 2022 ACS-NSQIP database was queried to identify adults with achalasia who underwent RAHM versus LHM. Patients in the RAHM group were matched using Coarsened Exact Matching with the LHM group on their preoperative characteristics. Chi-square and Fisher exact tests were used for categorical analysis, and Student’s *t*-test was used for continuous data analysis. 30-day outcomes, including mortality, morbidity, operative time, length of hospital stay along with reoperation and readmission rates were compared between the two groups in both aggregate and matched cohorts.

**Results:**

In the aggregate cohort, patients in the RAHM were older (55.21 ± 16.28 vs. 49.71 ± 12.19, *p* = 0.007) and had a higher percentage of male patients (66.3% vs.49.5%, *p* = 0.011), but otherwise there were no statistically significant differences in co-morbidities between the two groups. In the matched cohort, there were no statistically significant differences in the preoperative characteristics between the two groups. There were no mortalities in the cohorts. In the aggregate cohort, RAHM was associated with higher morbidity (7.5% vs. 1.5%, *p* = 0.009) and longer operative times (166.65 ± 67.33 vs. 124.06 ± 52.61, *p* < 0.001). Similar findings were confirmed in the matched cohort. Overall surgical site infection (SSI), reintubation, deep vein thrombosis (DVT), and myocardial infarction (MI) were also higher in the RAHM group, but it did not reach statistical significance. There was no difference in readmission and reoperation rates, or length of stay between the two groups.

**Conclusions:**

In this study, RAHM had similar short-term outcomes compared to LHM, but may be associated with higher overall morbidity and longer operative times.

**Graphical Abstract:**

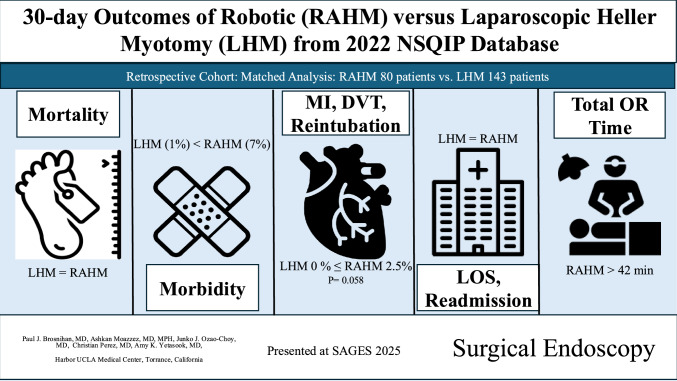

Achalasia, a rare disorder affecting esophageal motility and lower esophageal sphincter relaxation, produces a functional obstruction of the gastroesophageal junction. The most common etiology for achalasia is primary idiopathic achalasia that presents with progressive dysphagia, regurgitation, respiratory symptoms, chest pain, or weight loss. Since the early 2000s, minimally invasive Heller myotomy has been the standard treatment for esophageal achalasia, offering improved morbidity and mortality over open surgery [1–3]. Robotic-assisted Heller myotomy (RAHM) has gained popularity due to enhanced visualization, precision within the hiatus, and surgeon ergonomics. Prior studies suggested that RAHM reduces overall complications and rates of esophageal perforation compared to laparoscopic Heller myotomy (LHM), but at the expense of higher costs and longer operative times [4, 5]. However, given the rapid expansion of the robotic platform in recent years and the staggered learning curve of surgeons across the nation, there is limited data on recent prospectively collected 30-day perioperative outcomes. The objective of this study is to utilize a large database to evaluate the comparative 30-day outcomes of RAHM and LHM, including morbidity, mortality, readmissions, and operative times.

## Methods

### Study design and data collection

The study was approved by the Lundquist Institute at Harbor-UCLA, as a non-human subject study. Using the 2022 ACS NSQIP database, adult patients, ≥ 18 years, with a diagnosis of an achalasia (ICD10 codes: K22.0) who underwent an elective Heller myotomy (single procedure without additional CPT codes) were identified using CPT code (Minimally invasive Heller myotomy: 43,279). Patients who had the field “Robot used” as “Yes” were categorized as Robotic, and “No” as laparoscopic. This was a retrospective cohort study. Data included in the analysis were patients’ demographics, co-morbidities, operative time, and post-operative outcomes. Independent status was also assessed, and refers to a patient’s preoperative functional status, specifically their ability to perform activities of daily living without assistance. Dr. Ashkan Moazzez MD, MPH, did all statistical analysis for this project.

### Matching

To ensure comparability between the two study groups, patients undergoing RAHM were matched with those undergoing LHM using Coarsened Exact Matching (CEM) using 80 RAHM to 143 LHM. This statistical technique was employed to reduce confounding by creating a more balanced cohort while preserving a larger sample size. Preoperative characteristics used for matching included age and sex*.* These variables were selected based on their statistical difference in aggregate cohort and their potential influence on surgical outcomes and postoperative complications. Patients in the LHM group who lacked an appropriate match in the RHM group were excluded from the final matched analysis. Balance between groups was assessed using L1 Imbalance statistics of 0 in CEM match, and comparison of preoperative risk factors with no statistical difference after the match.

### Outcome measures

Following matching, statistical analyses were performed on both the aggregate and matched cohorts to compare perioperative and postoperative outcomes, including operative time, length of hospital stay, and complication rates. Primary outcomes included 30-day mortality and morbidity. Morbidity was defined as presence of any of the complications collected in NSQIP database. Secondary outcomes focused on operative time, reoperation and readmission rates. Statistical analyses included Chi-square and Fisher exact tests for categorical data and Student’s t test and ANOVA for continuous variables. SPSS, version 28.0 (IBM Corp, Armonk, NY), and 2-sided *P* < 0.05 was considered statistically significant.

## Results

Among 284 patients, 80 underwent RAHM and 204 underwent LHM. After matching, 80 patients in the RAHM group were matched with 143 patients in the LHM group on their preoperative characteristics. In the aggregate cohort, patients in the RAHM cohort were older (55.21 ± 16.28 vs. 49.71 ± 12.19, *p* = 0.007) and had a higher percentage of male patients (66.3% vs. 49.5%, *p* = 0.011), but otherwise there were no statistically significant differences in co-morbidities between the two groups in the aggregate cohort (Table 1). In the matched cohort, there were no statistically significant differences in the preoperative characteristics between the two groups (Table 1).

**Table 1:** Demographics in aggregate and matched cohorts by surgical approach

	Aggregate cohort			Matched cohort		
	RAHM n = 80 (%)	LHM n = 204 (%)	*P* value	RAHM n = 80 (%)	LHM n = 143 (%)	*P* value
Age (years)	55.21 16.28	49.71 17.19	0.007	55.21 16.28	53.40	0.423
Male (%)	53 (66.3)	100 (49.5)	0.011	53 (66.3)	91 (63.6)	0.696
White (%)	63 (80.8)	138 (81.7)	0.868	63 (80.8)	97 (82.9)	0.703
Hispanic (%)	11 (13.9)	17 (9.6)	0.306	11 (13.9)	11 (8.9)	0.259
BMI (Kg/m2)	27.65 7.01	27.42 6.22	0.393	27.65 7.01	27.65 6.13	0.500
ASA 3 and 4	39 (48.8)	92 (45.1)	0.579	39 (48.8)	71 (49.7)	0.897
Smoking	12 (15.0)	22 (10.8)	0.325	12 (15.0)	13 (9.1)	0.180
Diabetes	11 (13.8)	20 (9.8)	0.337	111 (13.8)	20 (14.0)	0.961
COPD	2 (2.5)	6 (2.9)	0.840	2 (2.5)	4 (2.8)	0.895
CHF	2 (2.5)	2 (1.0)	0.328	2 (2.5)	2 (1.4)	0.552
HTN	32 (40.0)	64 (31.4)	0.167	32 (40.0)	51 (35.7)	0.521
Steroid Hx	4 (5.0)	5 (2.5)	0.270	4 (5.0)	2 (2.1)	0.233
Bleeding disorder	1 (1.3)	3 (1.5)	0.887	1 (1.3)	2 (1.4)	0.926
ESRD	0 (0.0)	1 (0.5)	0.530	0 (0.0)	0 (0.0)	N/A
Preoperative ventilator	0 (0.0)	0 (0.0)	N/A	0 (0.0)	0 (0.0)	N/A
Ascites	0 (0.0)	0 (0.0)	N/A	0 (0.0)	0 (0.0)	N/A
Inpatient	48 (60)	132 (64.7)	0.459	48 (60)	99 (69.2)	0.163
Metastatic cancer	0 (0.0)	1 (0.5)	0.530	0 (0.0)	0 (0.0)	N/A
Elective	77 (96.3)	197 (96.6)	0.896	77 (96.3)	139 (97.2)	0.696
Preoperative transfusion	0 (0.0)	0 (0.0)	N/A	0 (0.0)	0 (0.0)	N/A
Preoperative sepsis	0 (0.0)	2 (1.0)	0.374	0 (0.0)	1 (0.7)	0.453
Preoperative covid	0 (0.0)	3 (1.5)	0.276	0 (0.0)	2 (1.4)	0.288
Post-operative covid	1 (1.3)	1 (0.5)	0.491	1 (1.3)	1 (0.7)	0.676
Transferred	0 (0.0)	5 (2.5)	0.158	0 (0.0)	3 (2.1)	0.192
Independent	75 (97.4)	200 (98.5)	0.528	75 (97.4)	141 (99.3)	0.250

In bivariate analysis, there were no mortalities. In the aggregate cohort, RAHM was associated with higher morbidity with OR 5.43 (95%CI 1.32–22.28, *p* = 0.019). This was also seen in the matched cohort with OR 5.71 (95% CI 1.12–29.02, *p* = 0.035).

In the aggregate cohort, overall surgical site infection (SSI) was statistically significant within the RAHM compared to LHM (2.5% vs. 0.0%, *p* = 0.023). However, in the matched cohort this only approached significance (2.5% vs. 0.0%, *p* = 0.058). Deep vein thrombosis (DVT) rates were significantly higher within the RAHM cohort in the aggregate cohort (2.5% vs. 0.0%, *p* = 0.023), however this only approached statistical significance in the matched cohort (2.5% vs. 0.0%, *p* < 0.058). This trend was also seen in myocardial infarction (MI); there were statistically significant rates in the aggregate cohort (2.5% vs. 0.0%, *p* < 0.023), but only approaching statistical significance in the matched cohort (2.5% vs. 0.0%, *p* < 0.059). Reintubation followed this pattern with statistically significant rates in the aggregate cohort (2.5% vs. 0.0%, *p* < 0.023) but only approaching statistical significance in the matched cohort. The rate of other complications were similar between the two groups (Table 2). There were no statistically significant instances of acute renal failure, stroke, urinary tract infection, transfusion, cardiac arrest, pulmonary embolism, sepsis, pneumonia, skin and soft tissue infection (superficial), skin and soft tissue infection (organ), or C. Difficile infection within the dataset.

**Table 2:** Postoperative outcomes of aggregate and matched cohorts by surgical approach

	Aggregate postoperative outcomes			Matched postoperative outcomes		
	RAHM n = 80 (%)	LHM n = 204 (%)	*P* value	RAHM N = 80 (%)	LHM n = 143 (%)	*P* value
Total operative time	166.65 ± 67.33	124.06 ± 52.613	< 0.001	166.65 ± 67.33	124.08 ± 56.026	< 0.001
Length of stay	1.80 ± 2.39	1.71 ± 1.85	0.379	1.71 ± 1.857	1.65 ± 1.836	0.809
Acute renal failure	1 (1.3)	0 (0.0)	0.110	1 (1.3)	0 (0.0)	0.180
Stroke	0 (0.0)	1 (0.5)	0.530	0 (0.0)	1 (0.7)	0.453
Myocardial infarction	2 (2.5)	0 (0.0)	0.023	2 (2.5)	0 (0.0)	0.058
Urinary tract infection	1 (1.3)	0 (0.0)	0.110	1 (1.3)	0 (0.0)	0.180
Transfusion	0 (0.0)	1 (0.5)	0.530	0 (0.0)	1 (0.7)	0.453
DVT	2 (2.5)	0 (0.0)	0.023	2 (2.5)	0 (0.0)	0.058
Cardiac arrest	0 (0.0)	0 (0.0)	N/A	0 (0.0)	0 (0.0)	N/A
Pulmonary embolism	1 (1.3)	0 (0.0)	0.110	1 (1.3)	0 (0.0)	0.180
Sepsis	1 (1.3)	0 (0.0)	0.110	1 (1.3)	0 (0.0)	0.768
Pneumonia	0 (0.0)	2 (1.0)	0.374	0 (0.0)	1 (0.7)	0.453
Reintubation	2 (2.5)	0 (0.0)	0.023	2 (2.5)	0 (0.0)	0.058
SSI (Any)	2 (2.5)	0 (0.0)	0.023	2 (2.5)	0 (0.0)	0.058
SSI (superficial)	1 (1.3)	0 (0.0)	0.110	1 (1.3)	0 (0.0)	0.180
SSI (organ)	1 (1.3)	0 (0.0)	0.110	1 (1.3)	0 (0.0)	0.180
C. diff	0 (0.0)	0 (0.0)	N/A	0 (0.0)	0 (0.0)	N/A
Morbidity	OR 5.43 (95%CI 0.28:3.10)		0.009	OR 5.71 (95%CI 0.11:3.36)		0.019
Readmission	OR 2.09 (95%CI-0.60:2.08)		0.270	OR 1.82 (95%CI-0.81:2.01)		0.396
Return to OR	OR 5.20 (95%CI-0.76:4.06)		0.136	OR 3.64 (95%CI-1.12:3.70)		0.263

When looking at operative times, RAHM had significantly higher total operative times (166.65 ± 67.33 vs. 124.06 ± 52.61, *p* = < 0.001) (Table 2). There was no difference in readmission rates in the aggregate cohort with an OR of 2.095 (95%CI-0.602:2.081, *p* = 0.280), compared to an OR of 1.82 (95% CI-0.81:2.01, *p* = 0.403) in the matched cohort. The reoperation rates and length of stay were low overall and equivalent in both the aggregate and matched cohorts (Table 2).

## Discussion

In this study, which provides a comparative analysis of RAHM versus LHM using the large prospectively collected NSQIP dataset, RAHM demonstrated equivalent mortality, reoperation and readmission rates as well as a similar length of hospital stay compared to LHM; however, RAHM was associated with higher overall morbidity and a longer average operative time.

Minimally invasive Heller myotomy has long been established as the preferred surgical approach for achalasia, with robust evidence supporting its safety and efficacy in achieving long-term dysphagia relief [3]. Our study aligns with existing literature demonstrating equivalent 30-day mortality between RAHM and LHM, consistent with prior cohort analyses and meta-analyses [3–6]. However, we observed a statistically significant increase in overall morbidity in the RAHM cohort, driven primarily by elevated rates of deep venous thrombosis (DVT), myocardial infarction (MI), and soft tissue infection (SSI), both in aggregate and matched analyses. This finding contrasts with prior large-scale studies, such as Chacko et al. in 2022, which reported improved morbidity in the robotic cohort, although notably, their analysis did not assess DVT or soft tissue infection rates, which may partially account for the discrepancy [6]. Conversely, the meta-analyses by Ataya et al. and Shaligram et al. reported no significant differences in morbidity or mortality but lacked detailed evaluation of individual postoperative complications [3, 4]. These comparisons highlight the importance of granular outcome analyses to uncover specific risks that may not be apparent in broader composite morbidity metrics.

A key strength of our study is that it is the first to utilize the NSQIP database following its 2022 update separating robotic procedures, providing a broad prospective national sample to examine postoperative complications. The divergence in morbidity we observed, particularly the trends toward significance in DVT, MI, and SSI within the matched cohorts, is notable, especially given the equivalence in baseline co-morbidities. These findings suggest that factors beyond patient characteristics, such as perioperative management and procedural nuances, may contribute meaningfully to complication rates and deserve further investigation. Among these potential contributors to increased morbidity, prolonged operative time emerges as a critical and potentially modifiable factor. With similar patient preoperative risk factors, the significantly longer operative duration in RAHM becomes a key differentiator. Prior NSQIP analyses, such as Sakran et al., have shown that surgeries exceeding 100 min increase venous thromboembolism (VTE) risk, with every additional 10 min elevating DVT risk by 7% [7]. While these associations are compelling, it is important to recognize that correlation does not imply causation, and we cannot exclude confounding factors such as surgical complexity or institutional protocols. Moreover, although measures such as preoperative chemical prophylaxis (e.g., heparin or enoxaparin) and intraoperative sequential compression devices (SCDs) are standard tools to mitigate VTE risk, the NSQIP dataset unfortunately does not track adherence to these prophylactic strategies, limiting our ability to assess whether compliance differed between RAHM and LHM cohorts [8, 9]. Additionally, the established correlation between prolonged operative time and increased SSI rates, as shown by Cheng et al., further reinforces the need to address operative efficiency in robotic surgery as a modifiable risk factor [10, 11].

The significant increase in operative time for RAHM, averaging 42 min longer than LHM, could partially explain the morbidity differences observed. Notably, this time difference exceeds the 23 min gap reported by Ataya et al. [4]. However, when compared to Milone et al., which found no statistically significant difference between platforms, or to Raja et al., a single-institutional high-volume center reporting a 21 min decrease in RAHM time, the discrepancies become striking [12, 13]. These variations suggest that operative efficiency may be highly dependent on institutional experience, surgical training, and robotic familiarity of the hospital staff.

A frequently cited advantage of RAHM is its potential to reduce intraoperative esophageal perforation, a complication with significant clinical consequences. While our dataset did not collect intraoperative data, we observed equivalent deep organ soft tissue infection rates, length of stay, readmission rate, and reoperation rates between RAHM and LHM. Previous studies have reported conflicting results regarding perforation risk. Chacko et al. found a statistically higher perforation rate, with RAHM using 2010–2015 data, whereas Ataya et al. and Aiolfi et al., reported a significantly lower perforation risk with robotic assistance, though both included Chacko et al.’s data in their analyses [4–6]. Our data suggest that, at minimum, perforation rates with RAHM are comparable to LHM, with minimal long-term complications, reinforcing the safety profile of the robotic approach.

There are several limitations in this study including NSQIP’s lack of granular data on preoperative factors (number or preoperative interventions, type of achalasia, manometry), or intraoperative complications (e.g., esophageal perforations, reoperative cases). Moreover, it does not capture functional outcomes (e.g., Eckardt scores, long-term dysphagia relief), or surgeon-level experience or institutional case volume which are central to evaluating the success of achalasia interventions. Finally, although we observed differences in operative time and morbidity, these may reflect unmeasured confounders, including institutional or surgeon-level variability, and the relatively small number of robotic cases raises the possibility of sampling bias.

## Conclusion

In this NSQIP-based study, RAHM had similar short-term outcomes compared to LHM, but was associated with higher overall morbidity including rates of MI, DVT, and SSI that approached significance, which may be related to longer operative times. These findings highlight the need for further investigation into surgeon proficiency, institutional factors, and patient selection in robotic esophageal surgery.

## Authors disclosures

Drs. Paul Brosnihan, Ashkhan Moazzez, Junko Ozao-Choy, and Christian Perez have no conflicts of interest. Dr. Amy Yetasook is a proctor for Intuitive.

## NSQIP disclosure

The American College of Surgeons National Surgical Quality Improvement Program and the hospitals participating in the ACS NSQIP are the source of the data used herein; they have not verified and are not responsible for the statistical validity of the data analysis or the conclusions derived by the authors.
